# The Use of Breath Analysis in the Management of Lung Cancer: Is It Ready for Primetime?

**DOI:** 10.3390/curroncol29100578

**Published:** 2022-09-30

**Authors:** Rachel J. Keogh, John C. Riches

**Affiliations:** Centre for Haemato-Oncology, Barts Cancer Institute, Queen Mary University of London, 3rd Floor John Vane Science Centre, Charterhouse Square, London EC1M 6BQ, UK

**Keywords:** breath analysis, lung cancer, volatile organic compounds, e-Nose technology, mass spectrometry

## Abstract

Breath analysis is a promising non-invasive method for the detection and management of lung cancer. Exhaled breath contains a complex mixture of volatile and non-volatile organic compounds that are produced as end-products of metabolism. Several studies have explored the patterns of these compounds and have postulated that a unique breath signature is emitted in the setting of lung cancer. Most studies have evaluated the use of gas chromatography and mass spectrometry to identify these unique breath signatures. With recent advances in the field of analytical chemistry and machine learning gaseous chemical sensing and identification devices have also been created to detect patterns of odorant molecules such as volatile organic compounds. These devices offer hope for a point-of-care test in the future. Several prospective studies have also explored the presence of specific genomic aberrations in the exhaled breath of patients with lung cancer as an alternative method for molecular analysis. Despite its potential, the use of breath analysis has largely been limited to translational research due to methodological issues, the lack of standardization or validation and the paucity of large multi-center studies. It is clear however that it offers a potentially non-invasive alternative to investigations such as tumor biopsy and blood sampling.

## 1. Introduction

Lung cancer is the most common cause of cancer-related mortality with approximately 1.8 million lung cancer-related deaths worldwide [1]. The stage at diagnosis significantly impacts overall prognosis. When diagnosed at its earliest stage, 88% of patients will survive for one year or more compared to approximately 19% of patients when diagnosed with stage IV disease [1]. Detecting lung cancer at an early stage can be difficult owing to the lack of clinical manifestations and reliable biomarkers. Additionally, lung cancer is a highly heterogeneous disease. It is classified according to histological subtype with non-small cell lung cancer (NSCLC) and small cell lung cancer (SCLC) accounting for approximately 85% and 15% of cases, respectively. With recent advancements in next-generation sequencing, NSCLC is further classified according to molecular subtype, and it is likely we will see more of this in the future with SCLC [2,3]. To date, tissue biopsy has remained the gold standard for diagnosis and management of various cancer types especially lung cancer. Tumor cells undergo metabolic changes in order to meet their high energy requirements for uncontrolled cell proliferation. Several oncogenic alterations can lead to changes in cancer cell metabolism. Exhaled breath offers an alternative method to capture these metabolic changes and genomic aberrations associated with lung cancer. 

Despite its promising ability to aid in cancer detection and management, its use has largely been limited to research. The biggest challenge to breath analysis is the lack of standardization in breath sample collection and the analytical methods used. Furthermore, most of the compounds present in breath are detectable at trace levels making it difficult to capture them with any technique currently available in clinical practice. Much of the research in this area has focused on lung cancer making this an exciting field. A simple “breath biopsy” offers a non-invasive method to analyze volatile compounds produced by metabolic processes. Endogenous compounds in exhaled breath such as organic compounds like nitric oxide as well as volatile organic compounds (VOCs) such as isoprene and acetone can be measured directly. Non-volatile compounds like isoprostane, leukotrienes and cytokines are measured in exhaled breath condensate and are present in exhaled breath as aerosol particles. Several analytical methods have been employed to detect both volatile and non-volatile organic compounds in breath. GC-MS remains the gold standard as it enables the identification and quantification of VOC composition at trace levels. However, it is time consuming, costly and requires technical expertise. The electronic nose has been a growing area of interest in recent years. This technology is designed to mimic the mammalian nose and detects specific volatile signatures known as “smell-prints”. 

The majority of biomarkers available in clinical practice for lung cancer rely on tissue and blood biopsies. Tumor histology is needed to characterize the subtype of lung cancer. It also remains the gold standard for molecular analysis however in certain cases blood biopsy such as for sensitizing EGFR mutations may be performed if tissue is unavailable. Other serum biomarkers are also in development to determine prognosis and monitor for treatment response [4,5,6]. Both tumor and blood samples can be difficult to obtain, time-consuming and may lead to significant patient distress. This review aims to summarize the history of breath analysis, provide an up-to-date overview of the current methodologies used for breath collection and analysis and explore the potential applications and limitations of breath biopsy in the future to help guide the treatment of patients with lung cancer.

## 2. Methods

We carried out a review of the literature for the current evidence for breath analysis as a method for detection, monitoring and assessment of treatment response in the management of lung cancer. We searched PubMed, and Embase databases for up to 1 June 2022 to identify studies focusing on breath analysis in the management of lung cancer with the search terms “lung cancer”, “breath analysis”. We focused on prospective studies by using the search terms “controlled study”, “diagnostic test accuracy study”, “prospective study”, ”pilot study”, ”case control study”, “cross sectional study” and “validation study”. We identified 114 articles on Embase and 76 articles on PubMed related to the use of breath analysis in the management of lung cancer that were suitable for inclusion. There is a significant amount of heterogeneity between studies due to the different sample collection, patient condition, environment and analysis methods used. 

**Figure 1:**
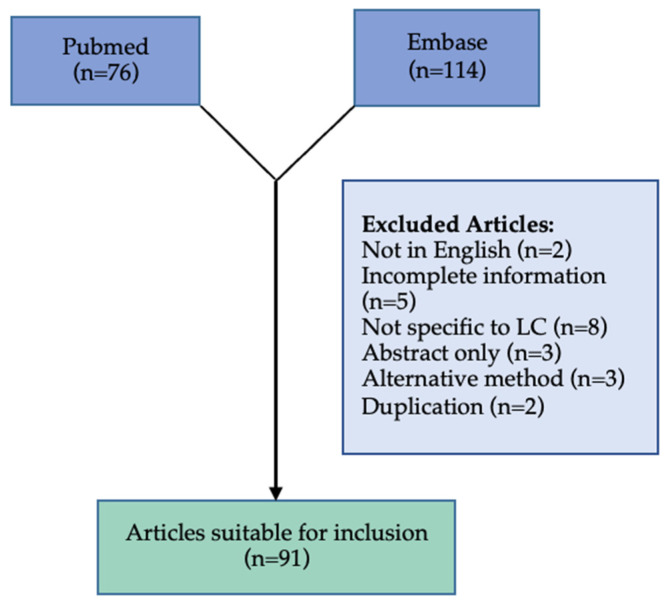
Flowchart of the systematic review process.

Articles were excluded for several reasons including: abstract only (n = 3), incomplete information (n = 5), not in English (n = 2), not specific to lung cancer (n = 8), technique for breath analysis not used (n = 3), duplication (n = 2). In total 91 articles were included (Figure 1).

## 3. History of Breath Analysis

The use of breath analysis and its association with disease dates back to the Ancient Greeks and Hippocrates who first described the terms “fetor hepaticus” and “fetor oris”. In 1949, Davidson and colleagues highlighted the potential utility of the composition of exhaled breath as potential indicators of disease in modern medicine when they demonstrated the presence of mercaptans in the breath of patients with severe liver disease [7]. They hypothesized that these molecules were the origins of the odor “fetor hepaticus”. The next big breakthrough in breath research came when Linus Pauling et al. identified over 200 different VOCs in exhaled air by gas chromatography [8]. In 1974, Riely and colleagues successfully identified and quantified small chain hydrocarbons, products of lipid peroxidation in exhaled breath [9]. They postulated that the concentration of these volatile hydrocarbons could potentially be used as biomarkers of in vivo oxidative stress. Phillips, a pioneer in the field of breath analysis, developed a method to detect acetaldehyde in breath and showed that this compound was produced endogenously within humans [10]. Through his research, he also demonstrated that the most common VOCs detected in breath were acetone, ethanol, isoprene, methanol, alkanes and several other alcohols [11]. In 1985, Gordon et al. reported that selected VOCs could be used to distinguish between 12 patients with lung cancer compared to 17 health controls using gas chromatography mass spectrometry (GC-MS) [12]. Although the sample size was small and there were a number of potentially confounding factors such as age and sex, this small case–control study highlighted the potential utility of breath analysis in the diagnosis of lung cancer. 

## 4. Composition of Exhaled Breath

Exhaled breath contains measurable levels of a host of compounds including proteins, lipids, DNA, microRNA such as bacteria and viruses. Generally, the components can be divided into three main groups: (i) inorganic compounds, e.g., carbon monoxide, nitrous oxide (ii) VOCs and (iii) non-volatile organic compounds. 

VOCs are the most extensively studied compound in exhaled breath in lung cancer (Table 1). They are classified according to chemical function into five main groups: aldehydes (e.g., heptanal, hexanal, decanal, nonanal, pentanal), ketones (e.g., acetone, 3-heptanone, 2-butanone, cyclohexane), alcohols (e.g., 2-ethylhexanol), hydrocarbons (e.g., 3-methylhexane, 4-methyloctane, 2,2-dimethyldecane) and aromatic compounds (e.g., 1,24-tri-methylbenzene, 1-methyl-4-(propan-2-yl)benzene, and p-xylene). The most common VOCs identified in breath are isoprene, acetone, ethanol, methanol and other alcohols [13]. 

**Figure 2:**
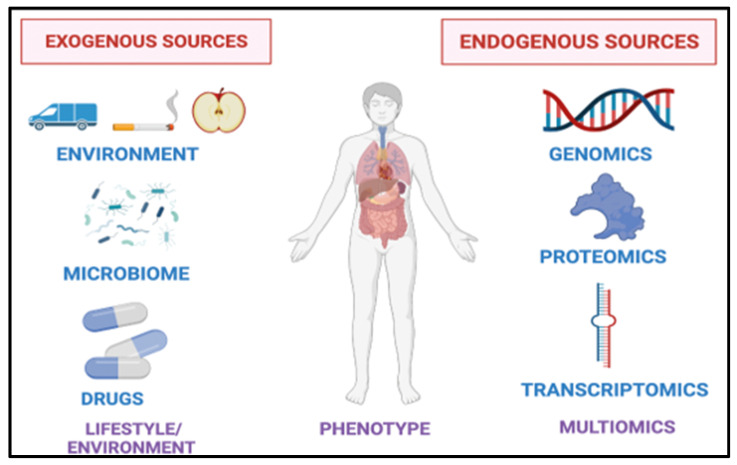
Sources of Breath Biomarkers. Exogenous sources such as diet, exercise, medications, microbiota and the environment influence the composition of breath. Endogenous sources such as our genes, proteins and RNA also play a role.

The clinical utility of VOCs in the diagnosis and management of a spectrum of diseases has been extensively studied [39,40,41,42]. VOCs are thought to be endogenous or produced as end-products of metabolism or exogenously from the environment (Figure 2) [43]. VOCs can also be metabolized and excreted through the liver and the kidney [44,45]. The clinical relevance of VOCs in the urine as lung cancer biomarkers has also been studied [46]. 

The origin of the various VOCs identified in different cancer types is not fully understood. Several VOCs are detectable in more than one cancer. For instance, aldehydes are associated with more than five tumor types [47]. Aldehydes are produced through different mechanisms including dietary intake, metabolized alcohols and smoking. They have been implicated in carcinogenesis by leading to DNA damage and preventing DNA repair and by oxidative stress [48]. Aromatic compounds such as benzenes have been identified in the exhaled breath of patients with lung cancer and may also be related to tobacco use. Nardi-Agmon et al. found an increase in the branched chain alkane, dodecane, in patients with advanced lung cancer who are no longer responding to treatment [37]. Hydrocarbons are primarily emitted in response to oxidative stress which plays a role in tumorigenesis. Wang et al. performed dynamic perioperative breath analysis and identified sixteen potential VOCs as lung cancer biomarkers including hydrocarbons and aldehydes [24].

Exhaled breath contains several non-volatile organic compounds that vary in composition from large organic molecules such as urea, organic acids and amino acids to proteins, surfactants and macromolecules. They are present in both physiological and pathological state and often reflect oxidative and nutritional stress (8-isoprostane, 3-nitrotyrosine and S-nitrosothiol) and inflammation (e.g., leukotrienes, histamine, prostaglandins) [49]. Carcinoembryonic antigen (CEA) is a glycoprotein that plays a role in cell adhesion and is normal produced in the gastrointestinal tissue during fetal development. Elevated CEA levels are present certain cancer types such as colorectal cancer. Zhang et al. used an immune-sensor to detect CEA in exhaled breath condensate (EBC) highlighting its potential use for disease monitoring in the future [50].

## 5. Breath Sample Collection

EBC contains approximately 99% water vapor as well as a fraction of respiratory airway lining fluid droplets [51,52]. There are various methods used for the collection and analysis of exhaled breath depending on the constituent of interest. If the target of interest is a lipid, protein, VOC, or microbiota then collection of EBC is generally the most suitable approach. Respiratory cells are not collected with EBC. Major issues surrounding the application of EBC in routine practice include the significant amount of intra-subject and inter-subject variability and the lack of standardization with regard to the collection and analytical methods used in clinical studies. In 2005, the American Thoracic Society/European Respiratory Society Task Force on Exhaled Breath Condensate published the first guidelines on methodological recommendations for the collection of breath samples [53].

### Breath Collection Devices

The use of breath analysis in the detection and management of cancer has been largely limited by the lack of standardization in breath sampling procedures and the need for repeatability. Several different breath sample collection devices are available.. The ideal breath sampling contained for clinical use is easy to use, durable, inert, and allows neither the inflow of environmental compounds nor the eflow of breath VOCs. The most commonly used breath collection apparatus consists of Tedlar^®^ bags which are composed of polyvinyl chloride, a chemically inert substance to most compounds in breath. A concern with Tedlar^®^ is the potential for cross-contamination, leaching and leaking [54]. Other polymer bags may also be used including Mylar and aluminum bags. VOCs from breath samples are then transferred into a sorbent tube for thermal desorption analysis with gas chromatography and a mass spectrometry detection (TD-GC/MS). The EBC can also be collected by subjects breathing through a tube inserted into a metal tube cooled to 0 degrees (R Tube™) or through a condenser (EcoScreen^®^) [55]. In this case, the subject breathes tidally for 10 to 20 min through the system and at the end of the time the condensate is collected and analyzed. 

The Bio-VOC™ breath sampler is an alternative breath collection apparatus that enables alveolar air to be collected which is felt to represent the deep air in the lungs. This is one of the advantages of this type of breath collection devices. Van den Velde et al. compared the differences in VOC composition between mouth and alveolar air in 40 subjects using the Bio-VOC™ breath sampler. A major limitation of the Bio-VOC™ sampler is the volume of air that is collected. The reported volume ranges from 88-150mls which is significantly lower than other collection devices [56,57,58,59,60,61,62].

In order to overcome the limitations of polymer bag sampling such as the need for sample storage and transport, more efficient sampling methods have been developed that allow for the collection of whole and/or alveolar breath directly on to a sorbent tube. An example of this include the Mistral breath sampler which samples only the end-tidal portion of the breath due to the volume control system [63]. The “Respiration Collection for In Vitro Analysis” or RECIVA (Owlstone Medical) has also been developed which collects both the whole and alveolar portions of exhaled air in function of specific parameters such as carbon dioxide [64]. It is also equipped with clean air during sampling to limit contamination with exogenous VOCs. The vast array of various collection devices highlights the need to standardize the process to ensure reproducibility. 

In the majority of studies, subjects inhale and exhale through the mouth during breath sample collection. In other studies, subjects were instructed to inhale through the nose and exhale through the mouth. Differences in both methods may influence mediators in EBC such as when air is humidified in the upper airways during nasal inhalation and mediators formed in the noses and the sinuses which are more likely to enter the lower airways during nasal inhalation. The ATS/ERS expert panel recommends the use of a nose clip during breath sample collection to avoid loss of sample through the nose and so inspiration bypasses the nose [53].

Within clinical studies evaluating EBC, techniques differ across steps such as the period of tidal breath prior to the breath sample collection, the technique used for breath collection, e.g., tidal breath sample, vital capacity, or alveolar breath sample or whether the inhaled air is filtered or unfiltered. This highlights the need for standardized procedural steps across studies if the use of breath analysis is going to progress into clinical practice. Depending on the biomarker, a range of analytical methods may be used for breath analysis including mass spectrometry, antibody-based assays, PCR and DNA or RNA sequencing. 

## 6. Methods of Breath Analysis

Two key analytical methods have been used to study VOCs in exhaled breath. This includes spectrometry-based methods to detect and quantify the chemical nature and composition of VOCs in any given sample. E-Nose technology, on the other hand, identifies patterns of VOCs that are termed “smell-prints”, identifying the overall pattern rather than specific VOCs. Another interesting approach previously studied is the used of dogs to detect VOCs. 

### 6.1. Spectrometry-Based Methods

Mass spectrometry (MS) has a wide range of clinical applications in proteomic, metabolomic, and drug pharmacokinetic studies. Several studies have been performed using mass spectrometry in the detection and management of lung cancer (Table 2). Coupling MS with separation systems such as liquid chromatography and gas chromatography improves the separation and identification of new biomarkers. VOCs are present in exhaled breath at trace concentrations (parts per billion or parts per trillion). As a result, the analytical method of GC-MS remains the gold standard for the identification and quantification of such compounds. Its use in clinical practice is limited due to the technically challenging pre-treatment steps, the technical expertise required and the time-consuming nature of this process. GC separates the components of the mixtures and then the MS defines the components structurally and allows for the identification of very small samples and strong structural analysis.

The principle of the IMS system is to separate ions in an inert gas under the influence of an electrical field. In contrast to GC-MS, this technique has a relatively compact design. The IMS system typically consists of three main systems: the ionization region, reaction region and the drift tube [65]. The most common ionization source is 63Ni which degrades analytes into ions. They then separate and move down a chamber at speeds relative to their size, geometry and mass hitting a Faraday plate at the end of the chamber [39]. As each ion hits the plate, an electrical signal is produced which when combined, produces an ion spectrum which is a fingerprint of the exhaled breath. Westhoff et al. used this technique to differentiate between 54 healthy subjects and 32 patients with lung cancer with 100% accuracy [66].

**Table 2:** Summary of clinical studies using e-Nose technology in lung cancer. Abbreviations: LC: lung cancer; NSCLC: non-small cell lung cancer; COPD: chronic obstructive pulmonary disease; IPF: idiopathic pulmonary fibrosis; PAH: pulmonary arterial hypertension; LD: lung disease; SAW: surface acoustic wave; MOS: metal oxide semiconductor; QMB: Quartz Microbalance.

Analytical Method	Number of Participants	Results	Ref.
Cyranose 320	14 LC patients, 19 *α*-1-anti-trypsin deficiency, 6 chronic pulmonary berryliosis, 20 HC	Sensitivity 71.4% Specificity 91.9% VOC signature: Isobutene, benzene methanol, ethanol, acetone, pentane, isoprene, isopranolol, dimethylsulfide, carbon disulfide, toluene	[67]
Colorimetric	49 NSCLC patients, 18 COPD, 15 IPF, 20 PAH, 21 HC	Sensitivity 73.3% Specificity 72.4%	[68]
Cyranose 320	10 LC subjects, 10 COPD (a), 10 HC (b)	Accuracy 85% Accuracy 90%	[69]
Nanosensor array with gold nanoparticles	30 LC, 26 colon cancer, 22 breast cancer, 18 prostate cancer, 22 HC		[70]
Quartz microbalance (LibraNose)	28 LC, 36 HC, 28 other lung disease	Sensitivity LC versus HC: 85%, LC vs. LC 92.8% Specificity LC versus HC 92.8%, LC vs. LD 78.6%	[71]
Colorimetric	92 NSCLC, 67 LC screening group, 70 indeterminate lung nodules	Sensitivity 70% Specificity 86%	[72]
Nanosensor array with single wall carbon nanotubes + gold nanoparticles	53 malignant nodules, 19 benign nodules	Sensitivity 86% Specificity 96%	[21]
LibraNose	42 LC, 18 HC	Accuracy 94%	[73]
SAW-based eNose	42 LC, 8 LD, 18 HC	11 VOCs predict LC (styrene, decane, isoprene, hexanal, propyl benzene, 1,2,4-trimethyl benzene, heptanal, methyl cyclopentane	[74]
MOS sensors-based eNose	43 LC, 58 HC	Sensitivity 95.3% Specificity 90.5% Accuracy 92.6%	[75]
SAW-based eNose	15 LC, 7 LD, 10 HC	11 VOCs predict LC	[76]
ENS Mk3 (E-Nose Pty, Sydney)	16 LC, 11 LD, 18 smokers, 11 ex-smokers, 33 non-smokers	*p*-values = 0.045, 0.025, 0.001 for discriminating based on different e-nose	[77]
Nanoscale NA-NOSE	25 LC (a), 22 HNC (b), 40 HC	Sensitivity 100% (a, b, c) Specificity 91% (a, b), 100% (c)	[78]
Colorimetric sensor assay	92 LC, 137 HC	Accuracy 81.1%	[72]
NA-NOSE with GC-MS	53 LC, 19 HC (a), adenocarcinoma and squamous (b), early and advanced disease (c)	Accuracy 88% (a, b, c)	[21]
MOS-SAW-based eNose	47 LC, 42 HC	Sensitivity 93.62%, Specificity 83.37%	[79]
Nanomaterial-based eNose	12 LC, 5 HC	Sensitivity 100% Specificity 80%	[80]
Cyranose 320	27 LC, 37 HC LC vs. Healthy smokers LC vs. never smokers	Sensitivity 63%, specificity 78%, accuracy 72% Sensitivity 96%, specificity 40%, accuracy 81%	[81]
QMB-based eNose	20 LC, 10 LD	Accuracy 90%	[82]
Cyranose 320	38 LC, 39 COPD	Sensitivity 80%, Accuracy 48%	[83]
SpiroNose	31 LC, 31 COPD (a), 37 asthma (b), 45 HC (c)	Accuracy: (a) 87% (b) 68% (c) 88%	[84]
Cyranose 320	25 LC, 166 current or former smokers without LC	Sensitivity 88%, Specificity 81.3%	[85]
BIONOTE	23 LC, 77 HC	Sensitivity 86%, Specificity 95%	[86]
Cyranose 320	165 LC, 335 total (91 non-cancer, 79 HC)	Sensitivity 87.3%, Specificity 71.2%	[87]
Carbon nanotube sensor array	56 LC, 188 HC	Sensitivity 75–100%, Specificity 86.2–96.6%, Accuracy 85.4–92.7%	[88]
Aeonose	144 LC, 146 HC	Sensitivity 94.4% Specificity 32.9%	[89]
MOS sensor array	6 LC, 10 HC	Sensitivity 85.7%, Specificity 100%	[42]
Cyranose 320	252 LC, 223 non-cancer controls (smokers (a), non-smokers (b))	Sensitivity 95.8%, specificity 92.3% Sensitivity 96.2%, specificity 90.6%	[90]

### 6.2. Electronic Nose (e-Nose) Technology

The electronic nose or e-nose is an instrument which comprises an array of electronic chemical sensors with partial specificity and an appropriate pattern recognition system that can recognize simple or complex odors [60]. e-Nose technology was developed to overcome the limitations of GC-MS including cost, portability, and the need for an expert operator. The e-Nose system is divided into three systems: (i) a sensor-array system (ii) a data processing system and (iii) pattern recognition system. The main element is the gas sensor array unit that is typically placed in a chamber with controlled humidity and temperature to detect VOCs in the exhaled air. The type of sensor used depends on the application and can include metal oxide semiconductor gas sensors, quartz crystal microbalance (QMS) sensors and surface acoustic wave (SAW) sensors. 

Recent advancements in the domains of electronics, analytical chemistry and artificial intelligence have resulted in an increase in the use of e-technology in healthcare. Several clinical studies have been conducted exploring the use of this method in the management of lung cancer (Table 2). Mc Williams used this method to demonstrate that breath profiling by e-nose technology may be used to differentiate between subjects with early-stage, potentially curable lung cancer matched with matched high-risk subjects without lung cancer [60]. They used the Cyranose 320 (Smith Detection Inc., Newark, CA, US) which is an e-nose system that consists of 32 polymer composite sensors. The statistical algorithms for pattern analysis include Principal Component Analysis (PCA) which decreases the initial dataset from 32 sensors to a set of 4 principal components that capture the greatest variance of the data [58].

### 6.3. Canine Detection

Dogs have a highly developed sense of smell and can detect smells up to threshold of several parts per trillion (ppt). The premise that dogs may be used to detect cancer was first postulated by Williams in 1989 who reported a case of a dog who showed an interest in a particular mole that was later identified to be melanoma [91]. Willis et al. published a study in 2004 which demonstrated that dogs could be trained to detect bladder cancer by sniffing urine samples [92]. Dogs were successfully able to identify cases of bladder cancer in 22 out of 54 (41%) of cases. Horvath et al. showed that dogs could successfully identify ovarian cancer from tissue and plasma with sensitivity and specificity of 100% and 95%, and 100% and 98%, respectively [93]. In 2006, McCulloch et al. successfully trained dogs to identify lung cancer with a sensitivity and specificity of 99%, respectively [94]. The use of canine scent detection is relatively inexpensive however it does require a significant amount of training and there is a paucity of high-quality studies published limiting its progress in the field. 

## 7. Factors Affecting Breath Analysis

### 7.1. Age/Sex

Several studies have demonstrated conflicting results in relation to the effect of age on breath analysis. Phillips et al. demonstrated a statistically significant difference in alkane contours between subjects aged 9 to 31 and 46 to 89 using GC-MS [95]. On the other hand, other groups have found no difference in breath analysis between age groups [68,69,96]. Dragonieri et al. found no significant difference in smell-prints using the Cyranose 320 in 20 subjects aged between <45 and >45 years old [69]. Wehinger et al. demonstrated no difference in VOC pattern across ages using PTR-MS [96]. Studies have also demonstrated that sex has no impact on the breath analysis [68,70,96].

### 7.2. Smoking

Studies have demonstrated that smoking can have an impact on breath analysis [20,41,97]. Gordon et al. explored the impact of smoking on VOC composition using GC-MS and found that cigarette smoking affected the VOC composition when comparing 5 smokers to 5 non-smokers [98]. However, the level of measured VOCs returned to baseline after fifteen minutes. Machado et al. found no difference between the breath analysis of non-smokers and current smokers in both healthy subjects and those with disease and instead concluded that any difference was likely related to the underlying pathological process rather than smoking status [67]. Mazzoni et al. also demonstrates no difference in colorimetry results based on smoking history (current, former, or non-smoker) [72]. Peng et al. also reported that participant’s smoking history did not impact on breath analysis [70].

Phillips and colleagues used GC-MS to examine the exhaled breath of subjects with lung cancer and subjects with a smoking history and a negative CT for lung cancer and developed a model to differentiate between the two cohorts [16,99]. The accuracy of the model was tested on an independent group of individuals. There was no difference in the ROC curves for current and former smokers. Fens et al. also demonstrated no difference in smell-prints between current and ex-smokers in a study using the Cyranose 320 to explore breath prints in patients with COPD and asthma [100]. 

In contrast, several studies have identified a link between specific VOCs and smoking [41]. Euler et al. reported increased levels of benzene and acetonitrile in the exhaled breath of smokers [101]. Although the level of benzene returns to normal soon after smoking, the level of acetonitrile can remain elevated for longer [102]. Wang et al. identified a difference in the levels of two VOCs (2,6,10,14-tetramethylpentadecane and 3,7-dimethyldecane) in the breath of smokers versus non-smokers highlighting the need to exclude these when development LC breath biomarkers [20]. It is essential to record the smoking habits of participants in clinical studies and it is advisable smokers refrain from smoking at least three hours prior to measurement to minimise the acute impact of smoking on mediator levels [53]. 

### 7.3. Pollution

Environmental factors such as air pollution can also impact the content of exhaled breath. A study by Lammers et al. identified different breath signatures based on the level of air pollution in the surrounding area including at the airport, highway and city centre [103]. A wide range of metabolites have been associated with air pollution largely due to oxidative stress and inflammation. Air pollution results in the upregulation of pro-inflammatory mediators, e.g., leukotrienes and downregulation of anti-inflammatory compounds, e.g., histidine. The link between air pollution and lung cancer development is of increasing clinical relevance. Studies have demonstrated that exposure to air pollution affects a patient’s survival [104,105]. Furthermore, recently published data suggests that increased exposure to ambient particulate matter < 2.5 μm increased the risk of lung cancer in non-smoking individuals with an EGFR mutation [106]. 

### 7.4. Food

The impact of food and nutrients on the constituents of breath is complex and can be divided into the acute effects of the fasted or fed state and the medium and long-term effects which are mediated by the changes in the gut microbiota. Smith et al. conducted a clinical study exploring the impact of several markers on breath after fasting and a protein-calorie meal in healthy subjects [107]. They found that breath acetone levels reached a maximum after fasting and then fell to a nadir four to five hours post-feeding. They demonstrated that the concentration of ammonia in breath was biphasic with a rapid fall to about 50% of the fasting level before rising two to three times their baseline at five hours after feeding. A slight increase in breath ethanol concentration was also observed, potentially related to the content of ethanol in some foods. Isoprene content did not differ significantly between the fed and fasting state. The ATS/ERS Task Force recommend that when measuring mediators affected by food or drink it is recommended to avoid this for a few hours prior to breath collection (for instance avoid caffeine if measuring adenosine) [53].

### 7.5. Medications

Medications may influence the mediator levels in EBC through a host of mechanisms. To be found in airway lining fluid, drugs need to diffuse across the alveolar capillary wall, the interstitial fluids, and the alveolar epithelial cells. Several drugs have been detected in exhaled breath including fentanyl, valproic acid as well as anesthetic agents such as propofol highlighting a patient use of breath analysis as a mean for real-time monitoring of drugs with a narrow therapeutics index [108]. An exploratory study by Borras et al. explored the impact of opioid analgesics on EBC [109]. The demonstrated that several volatile compounds such as normorphine and norhydromorphone and dihdyromorphone were detected in EBC. It is well-known that proton pump inhibitors such as lansoprazole affect the accuracy of the 14C-urea breath test by a pH-dependent mechanism used to test for Helicobacter Pylori [110]. A study evaluating the use of the ExaBreath^®^-device found that the antibiotics, piperacillin and meropenem, could be found and quantified in the exhaled breath of critically ill patients [111]. Although it did not correlate with plasma concentrations, it does suggest that performing therapeutic dose monitoring (TDM) of antibiotics through breath analysis may be possible in the future. Furthermore, exhaled breath may reflect ALF which can be difficult to obtain in critically ill patients who may not tolerate broncho-alveolar lavage for microbial analysis. 

### 7.6. The Microbiome

The microbiota of the gastrointestinal system varies greatly between individuals with people’s dietary habits accounting for approximately 20% of the structural variation [112]. Several studies have explored the influence of dietary fiber on the gut microbiota and on the basis of this, it has been postulated that the gut microbiota plays a key role in a number of bodily functions including modulating satiety, metabolism and enhancing gut-related immunity through mechanisms such as short-chain fatty acids (SCFA) and maintaining intestinal epithelial integrity [113]. Specific gut microbiome such as ruminococcaceae/faecalibacterium are associated with a favorable response to anti-PD-1 agents [114]. Neyrinck et al. conducted an exploratory study which found a correlation between faecalibacterium and specific breath volatile metabolites including butyric acid, butanol, acetaldehyde, ethanol and isoprene [115]. We postulate a potential use of breath analysis in the future may be in predicting treatment response by capturing the volatome associated with certain gut microbiota that may predict responders to immunotherapy.

### 7.7. Hypoxia

Studies have explored the potential impact of hypoxia on breath analysis. Harshmann et al. identified seven compounds (pentanal, 4-butyrolactone, 2-pentanone, 2-hexanone, 2-cyclo-penten-1-one,3-methylpentane and 2-heptanone) that were altered under hypoxic conditions in pilots using GC-MS [116]. Several VOCs were detected in the urine of trained participants undergoing 60 min of exercise on an ergometer at 70% of maximal oxygen uptake in order to elucidate the metabolic changes produced during rigorous exercise [117]. These included markers of lipid peroxidation such as aldehydes (propranol, butanal, pentanal, hexanal, heptanal, octanal, nonanal, malondialdehyde) and acetone. It is well-established that hypoxia plays a critical role in the tumor microenvironment and hypoxia is a poor prognostic factor in lung cancer [118]. It can lead to tumor progression through hypoxia-induced factor (HIF) which can lead to enhanced proliferation and angiogenesis. 

### 7.8. Other Diagnoses

#### 7.8.1. Lung Conditions

A number of breath biomarkers or VOCs are detectable in a range of lung conditions. Barker et al. demonstrated increased levels of several VOCs such as ethane, propane, benzene, and toluene in patients with cystic fibrosis [119]. Dragonieri et al. used an electronic nose to discriminate between patients with asthma and healthy controls and identified predominant VOCs in exhaled breath of patients with asthma including 4-methyloctane, 2,4-dimethylheptane, isopranolol, toluene, isoprene, alkane, acetic acid, acetone,11-trimethyl dodecane, 3,7-dimethyl undecane and 2,3-dimethyl heptane [69]. The potential impact of several mediators in exhaled breath have also been studied in COPD. Hydrogen peroxide is felt to be important in the pathogenesis of COPD and is likely related to oxidative stress. Nowak et al. found higher levels in EBC of patients with COPD compared to healthy controls [120]. Kostikas and colleagues also demonstrated that levels correlated with disease severity [121].

Breath studies have also been designed to detect several infectious diseases of the lung. Morozov and colleagues conducted a clinical study with the aim of developing non-invasive diagnostics for pulmonary TB [122]. This study involved 42 patients with TB and 13 healthy subjects and used exhaled microdroplets of lung fluid. Although this method had a relatively low sensitivity of 72% and specificity 58%, it did show promise for future development. The InspectIR COVID19 breathalyzer was the first COVID-19 breath test approved by the FDA [123]. This device uses GC-MS and was validated in a large study of 2409 participants and demonstrated a sensitivity of 91.2% and specificity of 99.3%. 

#### 7.8.2. Non-Pulmonary Conditions

Acetone is produced through dehydrogenation of isopropanol and the decarboxylation of acetoacetate. Patients with diabetes tend to have increased levels of ketone bodies, acetone, acetoacetic acid, and beta-hydroxybutyric acid in blood and urine. Breath acetone has been shown to correlate with the metabolic state in patients with diabetes [124]. Breath analysis may in the future offer an alternative to blood sampling for glucose monitoring for patients with diabetes mellitus. Several studies have also been performed on the impact of liver disease on exhaled breath. Several sulphur-containing compounds including dimethylsulfide, hydrogen sulfide and mercaptans are postulated to be biomarkers for liver disease [45]. Ferrandino et al. compared limolene levels in exhaled breath samples from 32 patients with cirrhosis, 12 patients with cirrhosis and HCC and 40 healthy controls [125]. They detected higher limolene levels in patients with cirrhosis. These studies also had small sample sizes and used various methodologies however it is clear that breath analysis shows promise in terms of its use in the management of a host of different conditions. 

#### 7.8.3. Extrapulmonary Malignancies

Several VOCs have been detected in the exhaled breath of other cancer types. Altomare et al. explored the potential utility of a breath test as a screening method for colorectal cancer using the RECIVA device in 83 patients with cancer and 90 healthy control [126]. They created a model using VOCS capable of discriminating between both with a true predictive value of 93%. Adam et al. found an increase in acetic acid, butyric acid and pentanoic acid in breath samples of patients with esophageal and gastric cancer [127]. The PAN-study: Pan-Cancer Early Detection Study (PAN) is an ongoing clinical trial aiming to recruit 1143 participants with the aim of differentiating between patients with and without different cancers by comparing breath biomarkers for a number of cancer types such as gastric, esophageal, and liver cancer [128].

## 8. Applications of Breath Biomarkers in Clinical Practice 

### 8.1. Early Detection and Diagnosis

Lung cancer screening by low dose computed tomography in high-risk individuals has been introduced in several countries including the United Kingdom. The screening population consists of those considered to be at high risk including those aged 55 to 74 years, who have a 30 pack year smoking history and have smoked for up to 15 years [129]. The National Lung Screening Trial (NLST) was the first randomized control trial that demonstrated a reduction in lung cancer-specific mortality through the introduction of low-dose CT screening [130]. One of the major limitations of this type of screening modality is the high rate of nodule detection that require CT follow-up resulting in increased cost and the potential need for biopsy or resection of a non-calcified nodule ultimately leading to significant patient anxiety. Breath analysis is a promising method to augment lung cancer screening with the aim of differentiating between cancer and benign findings. 

The altered genome and transcriptome during tumorigenesis and cancer progression results in dysregulated metabolic pathways and the build-up of aberrant metabolites. Cancer-derived volatile organic compounds as well as other metabolites can diffuse into the alveoli and be detected in exhaled breath. The LuCID study is a clinical trial recruiting up to 4000 patients referred with a clinical suspicion of lung cancer with the aim of identifying a VOC biomarker breath signature that can be used to distinguish between the presence and absence of lung cancer [131]. The results of this study are eagerly awaited and will hopefully give some clarity in terms of the clinical utility of breath biopsy in early detection across a larger study population. 

Handa et al. used ion mobility spectrometry to discriminate between individuals with lung cancer and healthy subjects [132]. They used VOC breath patterns and a decision tree algorithm to discriminate between different tumor histologies including adenocarcinoma, squamous cell carcinoma and small cell lung cancer. Furthermore, they also reported that increased D-dodecane may be useful in identifying EGFR mutated NSCLC (adenocarcinoma). Although the study population was small, this study highlights the potential value of breath analysis in classifying tumor type. 

### 8.2. Precision Medicine Applications

A significant proportion of patients with NSCLC present with alterations in specific genes that drive carcinogenesis. Next-generation sequencing has resulted in the detection of multiple distinct genomic aberrations leading to the development of biomarker-driven targeted therapies. Small diagnostic tissue sampling often leads to the failure of testing for validated oncogenic alterations in lung cancer. Smyth and colleagues demonstrated the potential feasibility of using EBC to detect the EGFR T790M mutations in patients with NSCLC [133]. Ryan et al. demonstrated that breath biopsy may be an effective alternative at detecting the presence of genomic alterations compared to tissue sampling [134]. They analyzed breath and blood biopsies for five genomic alterations (EGFR, PIK3CA, ERBB2, BRAF, KRAS) using ultrasensitive PCR in patients with lung cancer and compared results with corresponding diagnostic NGS tissue samples. The authors found a higher failure rate due to un-amplifiable DNA in the tissue samples compared to blood and breath. They also demonstrated higher numbers of mutations in EGFR, KRAS, PIK3CA in EBC and blood versus tissue NGS. This highlights a potential application of breath analysis as a non-invasive means to aid diagnosis and guide therapeutic decisions. 

Another interest method of capturing the metabolomic profile of tumors is through the Rapid Evaporative Ionization Mass Spectrometry (REIMS) coupled to an instantaneous surgical device known as iKNIFE. This offers an alternative method for metabolomic tracking resulting from specific genomic aberrations. This technique has shown great promise for being able to detect real-time cancer margins [135,136]. It does this by detecting the characteristic cancer signatures in the smoker emitted as a by-product of surgery. By building up a database, the iKNIFE can be used to predict the characteristic range of features that distinguish cancer according to the surgical aerosol. This also provides us with the opportunity to gain a greater understanding of the metabolic changes that underpin carcinogenesis. Koundouros et al. found that activated PIK3CA resulted in enhanced arachidonic acid-derived eicosanoids lead to uncontrolled cell proliferation using this method [137].

The detection and quantification of microRNAS (miRNAs) in exhaled breath condensate is poorly understood. Perez-Sanchez et al. conducted a clinical study performing genome-wide miRNA profiling and machine learning analysis on exhaled breath samples of 21 healthy controls and 21 LC patients [138]. They identified significantly altered levels of 12 miRNA in subjects with lung cancer. Dysregulated miRNA in EBC showed potential target genes related to LC carcinogenesis including CDKN2B, PTEN, TP53, BCL2, KRAS, EGFR highlighting the potential utility in the detection, stratification and monitoring of patients with LC. Several other small studies have demonstrated the potential value of microRNA in discriminating between health individuals and patients with LC [139,140]. The major limitation in bringing breath analysis to the clinic is the wide variability in study methodology and the very small sample sizes making it difficult to translate these results to a “real-world” LC population. Further large-scale studies are needed to explore whether any of these novel breath biomarkers could be used in the future to guide patient management. 

### 8.3. Monitoring for Treatment Response

Monitoring changes in the breath VOC signature before, during and after treatment could be used to assess treatment efficacy avoiding unnecessary ineffective treatment or leading to prompt discontinuation if a lack of treatment response is identified. Immune checkpoint inhibitors (ICIs) have revolutionized the treatment landscape of advanced NSCLC however a significant proportion of patients fail to respond to these novel therapies. Programmed cell death ligand-1 (PD-L1) as detected by immunohistochemistry (IHC) was the first validated biomarker approved to predict response to ICIs. This requires adequate tissue sampling which can pose significant challenges. Breath analysis offers a potentially cheaper more rapidly available method to predict ICI response. An observational study reported that e-nose technology could be used to predict which patients with advanced NSCLC may respond to anti-programmed cell death-1 (anti-PD-1) therapies [141]. The e-Nose technology correctly distinguished responders versus non-responders 85% of the time.

The development of a novel non-invasive method to monitor patients while on treatment so that ineffective therapy can be discontinued at the appropriate time is a key area of interest within the field of oncology. Buma et al. reported that e-Nose technology may also be used during ICI treatment to identify responders versus non-responders [142]. This is an interesting area that warrants further exploration given the potential difficulties in assessing ICI response through conventional imaging. “Pseudo-progression” during ICI therapy was first reported during a phase II trial of the anti-CTLA-4 agent, ipilimumab, in melanoma [143]. The authors described a patient who initially had an increase in the size of tumor lesions followed by a delayed response. This phenomenon is termed “pseudoprogression” and has subsequently been seen in a range of tumor types including bladder, breast, colorectal, esophageal and head and neck [144]. It poses a major clinical dilemma for physicians and can often result in the premature discontinuation of therapy. Identifying other biomarkers such as breath biopsy may provide clinicians with valuable information to guide decision-making. 

Nardi-Agmon et al. also conducted a clinical study enrolling 39 patients with advanced lung cancer to evaluate whether breath analysis could be used to guide treatment response [37]. They demonstrated that the presence of three VOCs could be used to identify patients with disease control (partial response/stable disease). They also used nanoarray to effectively identify patients who were no longer responding to therapy. 82% of patients had first line chemotherapy and 20% of patients had an EGFR inhibitor or ALK inhibitor indicating that breath analysis may be used to monitoring treatment response across a range of therapies. 

## 9. Conclusions

In summary, breath analysis has significant potential to aid in the screening, diagnosis and management of lung cancer. Despite this, further steps are needed for its use to become part of routine clinical practice. E-nose technology in particular holds great promise in translating breath biomarkers from the laboratory to the bedside. The critical steps needed to drive breath analysis forward include (i) the standardization of procedures for breath sample collection and analysis (ii) standardization of the instruments used to collect breath samples and (iii) standardization of reporting of and validating results. Integrating breath analysis into large, multi-center clinical trials with appropriate patient populations and control groups is essential. The results of the LuCID trial and PAN-study are eagerly awaited and will hopefully give us some more insight into the potential utility of breath biomarker in the detection of cancer in a larger study population. 

Lung cancer is characterized by a high degree of molecular heterogeneity associated with different mechanisms including genetic, non-genetic and epigenetic sources. The stage at diagnosis plays a central role in guiding patient prognosis and treatment plan. Finding better strategies to detect disease at an earlier stage is of the utmost importance and it is clear that breath analysis may be used in the future as an adjunct to low dose CT screening. At present, our current monitoring tools rely heavily on imaging or blood biomarkers to detect recurrence and guide treatment response. Developing innovative non-invasive tests that can be performed at the bedside could greatly assist clinical decision-making and spare patients’ unnecessary treatment. However, this technique has several limitations including the potential impact of compounding factors on results such as comorbidities, diet, medications, and the microbiome. As the fields of analytic chemistry and machine learning continue to advance, it is likely we will see further development in this area and potentially the application of non-invasive breath collection devices and analysis in routine clinical practice sparing patients invasive and costly procedures and treatment. 

## Figures and Tables

**Table 1:** Summary of clinical studies using Gas Chromatography/Mass Spectrometry (GC/MS) to explore VOCs in Lung Cancer. Abbreviations: LC: lung cancer; MLC: metastatic lung cancer; HC: healthy control; TNM: tumor node metastasis classification system; COPD: chronic obstructive pulmonary disease; PPV: positive predictive value; NPV: negative predictive value; PTR-MS: proton-transfer-reaction mass spectrometry; SPME: solid phase microextraction; FT-ICF-MS: Fournier transform-ion cyclotron resonance-mass spectrometry; SCLC: small cell lung cancer; NSCLC: non-small cell lung cancer, CA: cancer; ECC: exhaled carbonyl compounds, UA: upper airway; DA: distal airway; BPN: benign pulmonary nodules.; MAGIIC: mass abnormalities in gaseous ions and imaging correlates.

Analytical Method	No. of Subjects	Results	Ref.
GC-MS	12 LC subject, 17 controls	Increased levels of acetone, methyl ethyl ketone, n-propanol in LC patients compared to HC	[12]
GC-MS	87 patients with LC (67 patients had PLC, 15 patients had MLC), 91 patients had no evidence of LC, 41 HC	Sensitivity 90% (60/67), specificity 83% (34/41), cross-validation: sensitivity 85% (57/67), specificity 81% (33/41), smokers/ex-smokers had no effect on sensitivity, histology, TNM staging had no effect on specificity VOCs identified: butane, pentane, 5-methyl decane, 3-methyl tridecane, 7-methyl trodecane, 4-methyl octane, 2-methyl hexane	[14]
aGC-MS	NSCLC (pre- and post-surgery) = 36 and 24, respectively, 35 healthy smoker, 50 HC	Increased levels of isoprene, 2-methyl pentane in NSCLC vs. COPD cohort Reduced levels of toluene, heptane, benzene in NSCLC cohort vs. control smokers cohort Only isoprene decreased post-surgery	[15]
GC-MS	193 LC (128 prediction set, 65 test set); 211 HC (141 prediction set, 70 test set); 80 post-surgeries	Prediction of LC: Sensitivity 85%, specificity 80%, no difference between stage	[16]
SPME, GC on cell culture and breath analysis	29 LC, 13 HC, 7 chronic bronchitis	Prediction of LC: Sensitivity 86%, control specificity 69%, chronic bronchitis specifically 71%, PPV 80.6%, NPV 78%	[17]
PTR-MS + SPME GC-MS	220 LC (68 smokers,129 ex-smokers, 23 never smokers); 441 HC (84 smokers, 86 ex-smokers, 221 never smokers)	Decreased levels of isoprene, acetone, methanol in LC patients compared to controls (PTR-MS): 100% specificity For sensitivity: A:50% when add 2-butanone, benzaldehyde, 2,3-butanedione, 1-propranolol. B: 71% when add 3-hydroxy-2-butanone, 3-butyn-2-ol, 2-methyl-butane, 2-methyl-2-butene, acetophenone, 1-cyclopentene, methyl propyl sulphide, tetramethyl urea, n-pentanal, 1-methyl-1,33-cyclopentadiene, 2,3-dimethyl-2butanol. C sensitivity: 80% when add 1,2,3,4-tetrahydro-isoquinoline, 3,7-dimethyl-undecane, cyclobutyl-benzene, butyl acetate, ethylenimine, n-undecane	[18]
SPME + GC-MS	12 LC, 12 Healthy smokers, 12 Healthy never smokers	Higher levels of pentanal, hexanal, octanal, nonanal in LC patients vs. controls No significant difference between SCLC and NSCLC Pentanal: sensitivity 75%, specificity 95.8%	[19]
SPME, GC on cell culture and exhaled breath	85 LC, 70, benign lung disease, 88 HC	Significant difference in AUC > 0.6 and *p* < 0.01 in levels of 8-hexylpentadecane, 2-pentadecanone, 5-(1-methyl-)propylnonane, 3,7-dimethylpentadecanone between adenocarcinoma and squamous Correct classification of LC in 96.5% of cases, 34.3% of HC classified as benign and 33.3% of advanced LC incorrectly classified as early-stage LC	[20]
SPME + GC-MS	72 subjects with pulmonary nodules- 19 benign and 53 LC	Significant difference in 1-octene levels between benign and LC patients (*p* = 0.0486). No significant difference between stages and histologies.	[21]
TD-GC-MS	60 LC, 176 HC	Accuracy: 85 ± 4% Sensitivity: 83 ± 8% Specificity: 85 ± 7% AUC: 0.89 ± 0.06	[22]
SIFT-MS	148 LC, 168 HC	Accuracy: 0.92, Sensitivity: 0.96, Specificity: 0.88, AUC: 0.98	[23]
HPPI-TOFMS	157 LC, 368 HC	Accuracy 89.1%, Sensitivity 89.2%, Specificity 89.1%, AUC 0.952 VOCs: Acetaldehyde, 2-hydroxyacetaldehyde, isoprene, pentanal, butyric acid, toluene, 2,5-dimethylfuran, cyclohexanone, hexanal, heptanal, acetophenone, propylcyclohexane, octanal, nonanal, decanal, 2,3-dimethyldecane	[24]
Ion molecule reaction mass spectrometry	36 LC adenocarcinoma patients 25 squamous cell LC patients 52 colon cancer 45 HCC	Adenocarcinoma: Sensitivity: 86%, Specificity: 84% Squamous: Sensitivity 88%, Specificity 84% Colon: Sensitivity 96%, Specificity 73%	[25]
SPME and GC-MS	51 confirmed LC 38 with pathological findings suggestive of LC but not confirmed	CA+ versus HC: Accuracy 89%, AUC 0.94 CA- vs. HC: Accuracy 82%, AUC 0.906	[26]
GC-MS	108 LC patients 121 HC	Sensitivity: 80% Specificity 91.23%	[27]
TD-GC-MS	210 subjects in total (control group n = 89, COPD group n = 40, LC group n = 81)	Nanoic acid as biomarker for LC: Sensitivity: 32% Specificity: 88% PPV: 62% NPV: 67%	[28]
Silicon microchip Mass spectrometry	34 LC, 187 HC	Decrease in ECC after lung resection	[29]
GC-TOF-MS	48 LC, 130 Risk factor subjects (active smokers and ex-smokers), 61 HC (non-smokers without respiratory disease)	UA panel: LC versus RF: Sensitivity 58.1%, specificity 63.7% Control vs. LC Sensitivity 83.7%, Specificity 83.3% Control versus RF: Sensitivity 63.7%, Specificity 69.4% DA panel: LC versus RF: Sensitivity 75.5%, specificity 70.5% Control vs. LC: Sensitivity 77.5%, Specificity 89.8% Control versus RF: Sensitivity 79.5%, Specificity 71.4%	[30]
FT-ICR-MS	85 patients untreated LC, 34 BPN, 85 HC	Six carbonyl compounds (C_4_H_8_O, C_5_H_10_O, C_2_H_4_O_2_, C_4_H_8_O_2_, C_6_H_10_O_2_, C_9_H_16_O_2_) had significantly elevated concentrations in lung cancer patients vs. controls. LC versus benign nodules: Sensitivity 100%, Specificity 64% LC versus smokers: Sensitivity 100%, Specificity 86% LC versus non-smokers: Sensitivity 96%, Specificity 100%	[31]
SPME/GC-MS	138 subjects suspected of LC inc. 71 subsequently confirmed to have LC	AUC = 0.80, sensitivity 72.5% and specificity 75.8% at the flex point.	[32]
FT-ICR-MS	97 LC, 88 HC, 32 BPN	VOCs elevated in LC: 2-butanone, 2-hydroxyacetaldehyde, 3-hydroxy-2-butanone, 4-hydroxyhexanal Sensitivity 89.8% Specificity 81.3%	[33]
FT-ICR-MS	88 HC, 107 LC, 40 BPD, 7 solitary lung metastases	Four ECC elevated: 2-butanone, 3-hydroxy-2-butanone, 4-hydroxyhexanal, 2-hydroxyacetaldehyde Sensitivity 83% Specificity 74%	[34]
FT-ICR-MS	31 LC patients pre- and post-resection, 187 HC	Decrease in four ECCs post-surgery: 2-butanone, 3-hydroxy-2-butanone, 2-hydroxyacetaldehyde, 4-hydroxyhexanal	[35]
SPME-GC/MS	123 LC patients 361 HC	Sensitivity 63.5% Specificity 72.4% AUC 0.65	[36]
SPME-GC	13 HC, 29 LC patients, and 7 patients with chronic bronchitis	Sensitivity 86.2% Specificity 70% PPV 80.6% NPV 77.8% VOCs: Styrene, decane, isoprene, benzene, undecane, 1-hexene, hexanol, propyl benzene, 1,2,4-trimethyl benzene, heptanal, methyl cyclopentane	[17]
GC/MS, Na nose Sensor array	39 LC patients	VOCs: Styrene, *α*-Phellandrene (5-isopropyl-2-methyl-1,3-cyclohexadiene), dodecane,4-methyl PPV 86% Sensitivity 93% Specificity 85%	[37]
GC-MS	Discovery phase: 301 subjects screened for LC Validation phase: 161 subjects	MAGIIC biomarker (C4/C5 derivatives) LC Sensitivity 75.4% LC Specificity 85% LC Accuracy 84%	[38]
